# Meandering instability of air flow in a granular bed: self-similarity and fluid-solid duality

**DOI:** 10.1038/srep38457

**Published:** 2016-12-12

**Authors:** Yuki Yoshimura, Yui Yagisawa, Ko Okumura

**Affiliations:** 1 Department of Physics and Soft Matter Center, Ochanomizu University, 2–1–1, Otsuka, Bunkyo-ku, Tokyo 112-8610, Japan

## Abstract

Meandering instability is familiar to everyone through river meandering or small rivulets of rain flowing down a windshield. However, its physical understanding is still premature, although it could inspire researchers in various fields, such as nonlinear science, fluid mechanics and geophysics, to resolve their long-standing problems. Here, we perform a small-scale experiment in which air flow is created in a thin granular bed to successfully find a meandering regime, together with other remarkable fluidized regimes, such as a turbulent regime. We discover that phase diagrams of the flow regimes for different types of grains can be universally presented as functions of the flow rate and the granular-bed thickness when the two quantities are properly renormalized. We further reveal that the meandering shapes are self-similar as was shown for meandering rivers. The experimental findings are explained by theory, with elucidating the physics. The theory is based on force balance, a minimum-dissipation principle, and a linear-instability analysis of a continuum equation that takes into account the fluid-solid duality, i.e., the existence of fluidized and solidified regions of grains along the meandering path. The present results provide fruitful links to related issues in various fields, including fluidized bed reactors in industry.

Fluid flows in a medium often display spectacular instabilities; there are various examples of this in cosmological^1^, geophysical^2^, biological^3^, and physical systems^4^. Among such phenomena, meandering instability is familiar to everyone in the form of small rivulets of rain flowing down a windshield or, in a more large-scale form, in river meandering. In particular, for a meandering fluid interacting with a solid surface, the physics has been relatively well understood. The meandering of a liquid jet flowing down an inclined plate and some variations of this have actively been studied^5^,^6^,^7^ (for larger-scale experiments, see ref. 8 and references therein). For such a meandering, the physical mechanism has been elucidated in terms of force balance, linear stability^9^,^10^,^11^ and flow-rate fluctuation^12^. On the contrary, for a meandering fluid interacting with a surrounding “complex fluid,” the mechanism of the instability has yet to be clarified. This latter case includes the meandering of rivers, which is ubiquitous and is even found on Mars^13^, and has been studied in geography^8^,^14^, biology^15^,^16^,^17^, hydrodynamics^18^,^19^,^20^, and physics, from various viewpoints such as pattern formation^20^,^21^, random walks^22^,^23^, and statistical models^24^,^25^. For example, more than a half-century ago, shapes of meandering rivers were shown to be scale invariant or self-similar, i.e., the curvature, amplitude, and wavelength of the shape all scale with the width, using the data obtained by field studies^26^, which has yet to be understood. More recently, a systematic dependence of the sinuosity of meandering rivers on the Froude number has been shown, which is interpreted through results obtained by a simple numerical model^27^.

Here, we perform a small-scale experiment in which an air flow is created in a granular bed as follows: the flow of a lighter fluid is surrounded by a heavier “complex fluid,” which is similar to river meandering. As a result, we find that the flow can be destabilized to show meandering shapes. The meandering regime can be universally demonstrated as a function of the normalized flow rate and normalized granular-bed thickness. In addition, the meandering flow shape is found to be scale invariant or self-similar, as was shown for river meandering. These experimental findings can be explained by the proposed physical principles.

## Results

### Experiment

#### Setup

**Figure 1:**
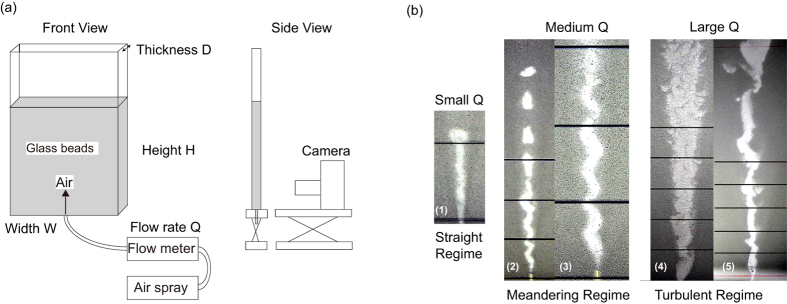
(**a**) Experimental setup. (**b**) Flow-shape change with the flow rate *Q*. Snapshots in (1–5) are obtained for the glass beads Bz01 for the cell width *W* = 80 mm under the conditions, (*D* [mm], *Q* [*μ*m^3^/s]) = (1.0, 0.5), (1.0, 1.25), (1.0, 1.17), (0.7, 1.67), and (1.2, 4.17).

The main setup consists of a transparent acrylic cell and a gas-flow controller (see Fig. 1(a)). The inside thickness *D*, width *W* and height *H* of the cell satisfy the condition 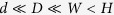. The cell was filled with beads with diameter *d* and connected at the bottom with a Teflon tube (F8006, Flon Industry, Japan) to the gas-flow controller (LogMIX, Front, Japan). Through the controller, “air” (nitrogen gas of density *ρ*_*A*_ = 1.29 g/cm^3^; A3125N, Kenis) was injected into the cell at the bottom at a fixed flux *Q*, while the cell was held in the upright position. The depth of the observed region in the granular layer was more than approximately 10 cm from the air-granular interface so that the granular pressure approaches a constant value due to Janssen’s mechanism^28^. The cell width *W* is either *W* = 80 mm or *W* = 40 mm. We used the following three types of beads: (1) glass beads with an average diameter *d* = 113 *μ*m (Bz01, As One), (2) glass beads with an average diameter *d* = 196 *μ*m (Bz02, As One), and (3) Alumina beads with an average diameter *d* = 124 *μ*m (Taimei Chemicals). The density of glass beads is *ρ*_*G*_ = 2.5 g/cm^3^ and that of alumina beads is *ρ*_*G*_ = 3.9 g/cm^3^.

#### Flow Regimes

**Figure 2:**
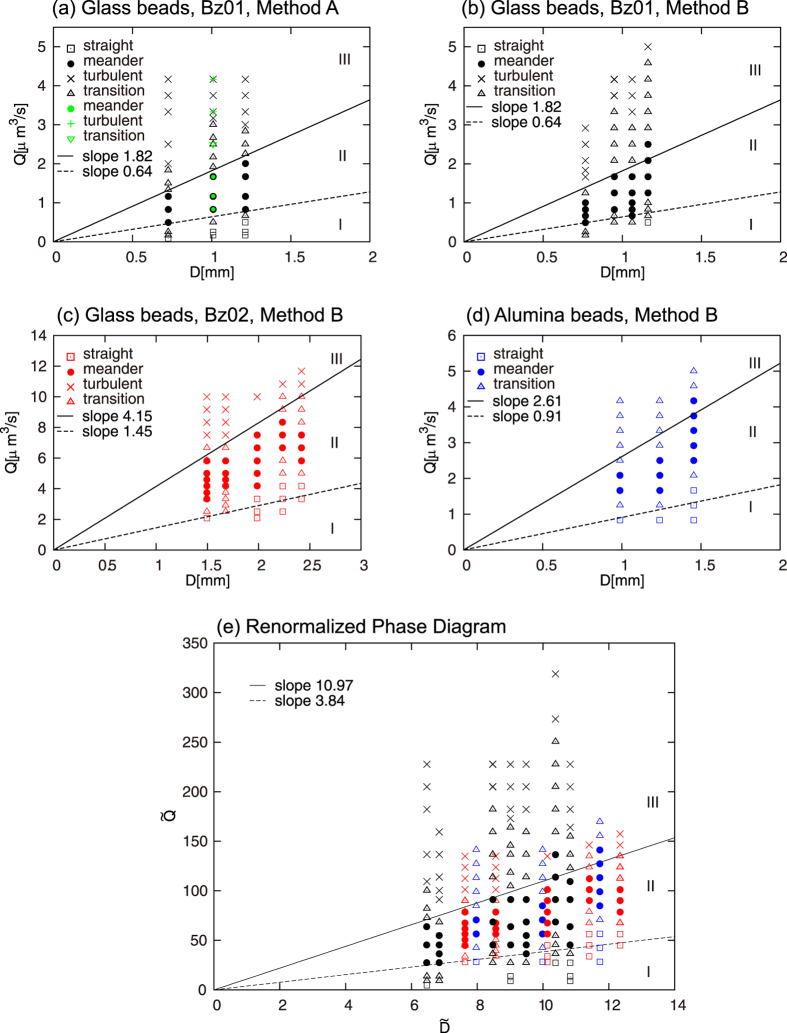
(**a**–**d**) Phase diagrams, *D* vs. *Q*, for different beads and packing methods. The green symbols in (**a**) are the data obtained for *W* = 40 mm. All of the other data are obtained for *W* = 80 mm. (**e**) Phase diagram with renormalized axes. The labels, I, II, and III, represent the straight, meandering, and turbulent regimes, respectively.

**Figure 3:**
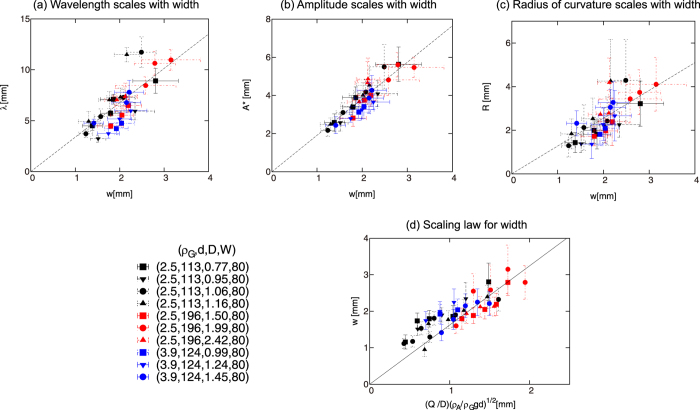
(**a**–**c**) Wavelength *λ*, “amplitude” A*, and radius of curvature *R* vs. width *w*, demonstrating that the three quantities are all linearly dependent on *w*. For the definition of A* see Fig. 4(b) and Methods. (**d**) *w* vs. renormalized *Q*, demonstrating a scaling law for *w*. The units of *ρ_G_, d, D,* and *W* are g/cm^3^, µm, mm, and mm, respectively.

**Figure 4:**
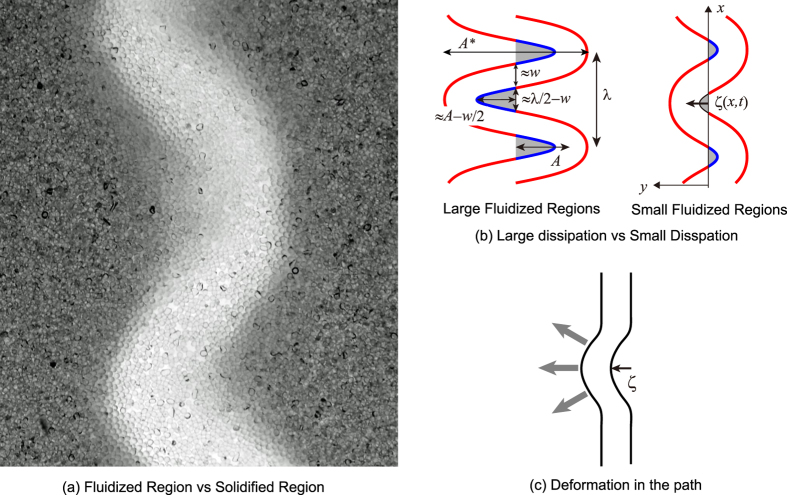
(**a**) Visualization of fluidized and solidified regions via a snapshot taken with a relatively long exposure time, in which the grains in the fluidized region move but the grains in the solidified region do not. (**b**) Illustration of wavy paths with large and small fluidized triangles. (**c**) Illustration of a path with a small perturbation.

The flow created in the glass-bead bed is divided into three regimes, as shown in Fig. 1(b) (movies for Bz01 are available as Supplementary Movies 1–3). (I) Straight regime: a nearly straight path ends in the middle of the granular bed as a result of absorption of air by the bed. (II) Meandering regime: regular wavy shapes appear in a transient but well-defined manner; above the wavy path, bubbles are sometimes formed. (The observed meanders appear near the outlet of the tube before they slightly travel along the path; once stabilized the meander shapes do not travel.) (III) Turbulent regime: different from the other regimes, the path goes through the bed to the air-granular interface, and the flow appears as a hydrodynamic turbulent flow. The turbulent flow tends to be straight for small *D* (Fig. 1(b)–(4)) with fingering patterns along the side edges of the path, whereas the flow tends to be wavy for large *D* (Fig. 1(b)–(5)). Regimes I to III appear in this order as the flow rate *Q* increases for a given set of *D*, *d*, and *ρ*_*G*_.

#### Phase Diagram

In Fig. 2(a–d), the phase diagrams are shown on the (*D*, *Q*) space. The filled circles stand for the case in which the path is clearly in the meandering regime (at least 4 waves can be recognized). The open squares (crosses) stand for the case in which the path shape is clearly in the straight (turbulent) regime. The open triangles represent the case in the crossover region in which the waves are less than three and the path has characteristics of more than two regimes. All the ensuing analyses of the meandering path are performed for the data represented by the filed circles in Fig. 2.

The two results for the glass beads, Bz01, shown in (a) and (b), demonstrate a weak dependence on the way of packing the beads in the cell. We obtained (a) through the following process (Method A): (1) we poured the beads in the cell, (2) injected a strong gas flow of approximately *Q* = 5 *μ*m^3^/*s* and (3) gradually decreased the flow to record the data points. In contrast, to obtain (b), we added one more step (Method B): between step (1) and (2), we injected a strong gas flow of approximately *Q* = 5 *μ*m^3^/*s* and gradually decreased the flow to zero. In spite of this difference, the results in (a) and (b) look reasonably similar to each other. In fact, the slight differences in (a) and (b) may instead be due to differences in other conditions that cannot be controlled precisely, such as humidity and electrostatic effects. In the present study, all the data, except for the data in (a), are obtained through Method B.

The phase diagrams in Fig. 2(a–d), obtained for glass beads, Bz01 and Bz02, and the alumina beads, look similar to one another. To quantify the similarity, we collect in plot (e) all the data shown in (a)–(d), preserving the colors and symbols in the phase diagram with renormalized axes, *D*/*d* and *Q*/*Q*_0_ (the definition of *Q*_0_ will be given later). The dashed and solid lines in Fig. 2(e) are drawn according to the following principles: we consider the slopes of the lines obtained by connecting the origin with all the points represented by filled circles and select the line with the smallest (largest) slope as the dashed (solid) line (in selecting the solid line, we neglect two exceptional filled circles at 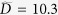 and 11.7).

In Fig. 2(e), we clearly see universal or robust features of the meandering phenomenon in the present study: the dashed and solid lines in Fig. 2(e), which are selected as specified above, work reasonably well as the lower and upper phase boundaries, respectively, irrespective of *d* and *ρ*_*G*_. In fact, when the dashed and solid lines in (e) are mapped back to the original plots, (a)–(d), the corresponding dashed and solid lines in (a)–(d), work well as guides for the eyes to recognize the phase boundaries in each plot. Note that the triangles corresponds to an intermediate state, i.e., a path with characteristics of a meandering path. Further remarks will be given in the Discussion.

In Fig. 2(a), it is shown that the change in the cell width *W* has practically no effect if the condition 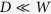 is satisfied. The symbols in green are the data obtained with a cell of width *W* = 40 mm. The green data nearly overlap with the data obtained with a cell of width *W* = 80 mm.

#### Self-similarity

The centroid of the meandering shape is, in principle, of the form *x* = *A* sin(2*πy*/*λ*), with amplitude *A* and wave length *λ*, where *x* and *y* are the vertical and horizontal positions within the cell, respectively. If this description is essentially correct, the curvature *R* will satisfy the scaling law


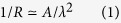


As shown in Fig. 3(a–c), we found that the three characteristic lengths all scale with the width *w*. From numerical fitting, we obtain


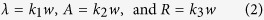


with *k*_1_ = 3.31 ± 0.13, *k*_2_ = 0.445 ± 0.02 and *k*_3_ = 1.27 ± 0.05. These relations are summarized as the following scaling laws:


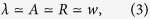


which means that the meandering shape is scale-invariant or self-similar: there is a single length that characterizes all different meandering shapes.

#### Scaling law and renormalization

As shown in Fig. 3(d), we experimentally found that the width *w* is well characterized by the following scaling law involving the gravitational acceleration *g*:


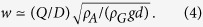


Here, as indicated in Fig. 3(d), the numerical coefficient is of the order of unity, as expected. A simple physical explanation of this scaling law will be given later.

The renormalization of the two axes performed for the phase diagram in Fig. 2(e) is in fact motivated by this scaling law, which can be cast into the form,


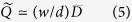


with 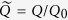 and 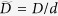. The characteristic flow rate is given by


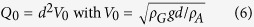


Here, as will become clear shortly, *V*_0_ scales with the velocity of the air flow in the granular medium, which implies that *Q*_0_ is the flow rate for a section 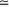
*d*^2^. In other words, the renormalization of *D* and *Q* employed in Fig. 2(e) corresponds to taking *d* as the unit length.

Equation (5) suggests that the dashed and solid lines (phase boundaries) in Fig. 2(e) correspond to the lower and upper bounds for the width *w* in the length unit *d*. This is because on the 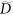-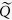 plot, the data on a straight line going through the origin are the collection of data having the same value of *w*/*d*. This implies that the slopes of the dashed and solid lines correspond to the minimum and maximum values of *w* in the unit *d*.

#### Fluid-Solid Duality

As shown in Fig. 4(a) the granular medium surrounding the meandering path is categorized into fluidized and solidified regions. This snapshot is taken with a relatively long exposure time, 1/20 sec, so that we can recognize regions in which particles are almost at rest during this exposure time (solidified region) and regions of the opposite character in which particles are moving during the same exposure time (fluidized region). Quite naturally, solidified regions are found near “convex” interfaces (“convex” when seen from the side of the air path) because the air must be pushed back by the interface to change its direction of flow (as a reaction, the air flow pushes the interface through a centrifugal force). Fluidized regions are found near concave interfaces, which is also natural because of the absence of centrifugal force near the convex interface that, if it existed, would push the interface. In addition, it is potentially noticeable in the snapshot that the density in the fluidized region is lower. This suggests that particles lose contacts with one another in the region, that is, the medium is fluidized. These points are more clearly visible in a movie taken by a high-speed camera (see Supplementary Movie 3). Note that the area of the fluidized region is rather limited because of the principle of minimum dissipation discussed below (in the ideal case, the area of the fluidized region would be zero within the crudest approximation). Because of this, the duality is not necessarily easy to recognize in Fig. 4(a) but is far more clearly visualized in the movie, Supplementary Movie 3. The reader is strongly encouraged to view this movie to confirm the distinction between the solidified and fluidized regions.

### Theory

#### Principle of minimum dissipation

The self-similarity of the meandering shape, described by the scaling laws in Eq. (2), can be explained as a result of the principle of minimum energy dissipation in the granular medium. Since the fluidized and solidified regions are associated with the concave and convex parts, respectively, the fluidized regions can be recognized as the triangular parts shown in gray in Fig. 4(b). In these regions, energy is predominantly dissipated due to inelastic collisions between particles: the area of a triangular region is a measure of energy dissipation. The area scales as (*A* − *w*/2) (*λ*/2 − *w*)/2, as illustrated in 4(b). This decreases to zero as the set (*A*, *λ*) approaches the following value:


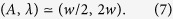


In other words, within this crude approximation, the area of the fluidized region is optimized to zero. Accordingly, the fluidized region is in fact very limited. (This is the reason the distinction between the solidified and fluidized regions are not necessarily easy to recognize in a still snapshot, but easy to recognize in a movie, as mentioned above).

In fact, by inspection, we confirm that all three snapshots of meandering paths in Fig. 1(b)-(2) and (3) and 4(a) nearly satisfy this condition! No paths resemble the path shown in the left panel of Fig. 4(b) with large triangles; rather the path resemble the path shown in the right panel of Fig. 4(b), with small triangles whose areas are almost zero. At a quantitative level, we can confirm that Eq. (7) implies 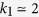 and 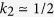, which compare quite well with the experimentally observed values given below Eq. (2).

Equation (7), which is realized reasonably well in the experiments, expresses that the dissipation is minimized when the scaling relation 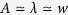 holds. These scaling laws are combined with Eq. (1) to obtain the relation 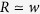. Therefore, we confirm Eq. (3) on the basis of the principle of minimum dissipation.

#### Principle of the floating ping-pong ball

The width *w* of the meandering path is essentially determined by the principle of the floating ping-pong ball^29^, a familiar phenomenon in which a ping-pong ball is levitated with air flow from a blower, which is explained by Bernoulli’s Principle. In the present case, at the top end of the air path, as shown in the snapshot in Fig. 1(b)-(1) and (2), the sand particles are floating, similarly to a floating ping-pong ball. At the level of scaling laws, this condition is expressed as the balance between the dynamic pressure of the air flow of velocity *V* and the gravitational force acting on a sand particle, i.e., 
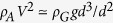
. This relation, which in fact means that *V* scales as *V*_0_, which was already defined in Eq. (6), is combined with the condition of the flow conservation,


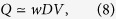


to reveal the scaling law for the width given in Eq. (4).

As shown in Fig. 3(d), the scaling law is reasonably well satisfied for the meandering paths. This implies that, in the present experimental conditions, the permeation of air in the granular medium is not significant at the level of scaling laws. This is because the scaling law in Eq. (4), which is confirmed in Fig. 3(d), is derived with the assumption of the flow conservation given in Eq. (8).

#### Minimal model for fluid-solid duality and linear instability

The observed meandering instability can be understood on the basis of the two-dimensional Navier-Stokes equation for the air flow. Since the Reynolds number of the flow is relatively high, the distribution of the flow speed on the section perpendicular to the flow is almost homogeneous, except near the boundaries (i.e., the flow is a quasi plug flow). As a result, the dynamics are well described by considering a path of linear density *ρ*_*A*_*wD* flowing with velocity **u**(*x*, *y*, *t*) = (*u*, *v*), with air density *ρ*_*A*_^10^,^30^,^31^.

Equation (4), which has already been explained in terms of the principle of the floating ping-pong ball, can also be explained in this framework. When the path is nearly straight, the dominant component of the velocity is *u* 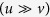 and its stationary dynamics is described simply by *ρ*_*A*_*uu*_*x*_ + *p*_*x*_ = 0 (the subscripts denote partial derivatives and *p* is the isotropic pressure) because viscosity and gravity for the air flow can be neglected; the quantity *ρ*_*A*_*u*^2^/2 + *p* is preserved along the path (Bernoulli’s principle). Throughout most of the path, this quantity scales as *ρ*_*A*_*V*^2^/2 + *p*_0_, where *V* is the velocity along the path and *p*_0_ is the atmospheric pressure. However, it scales as *p*_*G*_ + *p*_0_ near the top end of the path: at the top of the path, as suggested above, granular particles are fluidized with a high packing fraction, giving a pressure scale *p*_*G*_ of the order of *ρ*_*G*_*gd*^3^/*d*^2^. Balancing the two pressure scales, we obtain Eq. (4).

The initiation of the meandering instability can be understood from the *y* component of the equation of motion for the path of linear density *ρ*_*A*_*wD*, where a deformed path shape is described by *y* = *ζ*(*x*, *t*) (see Fig. 4(c)). This equation of motion may be described by the equation that minimally reflects the fluid-solid duality of granular materials along the meandering path as illustrated in Fig. 4(a,b):


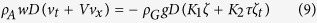


Here, *V* is the air-flow velocity along the path. The left-hand side of this equation stands for inertia, while the right-hand side expresses a simple and minimal interaction of the flow with the granular medium for the instability: the *K*_1_ term expresses an elastic response, characterizing the solid-like property, whereas the *K*_2_ term represents a viscous response, characterizing the liquid-like property, with the subscript *t* denoting the time derivative. Here, *K*_1_ and *K*_2_ are numerical coefficients of the order of unity, whereas *τ* is a characteristic time.

##### Maximum wavelength for instability

The linear stability analysis of Eq. (9) leads to two conditions for the instability, with the one determining the maximum wavelength for the instability. By seeking the solution to a linearized version of Eq. (9) of the form 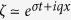 with *q* = 2*π*/*λ*, we find that the solution becomes unstable (Re[*σ*] > 0) when the following two instability conditions are both satisfied:


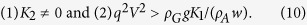


The first condition implies that the instability requires the *K*_2_ term. The second condition can be expressed as 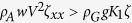; the centrifugal force overcoming the restoring force leads to the instability. This is considerably different from the meandering instability of rivulets interacting with solid plates^10^, in which the centrifugal force does not play a role in triggering the instability. The second condition also defines the maximum wavelength *λ*_*m*_ for unstable modes:


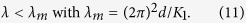


##### Fastest growing mode

The scaling law 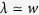 in Eq. (3), which has already been justified on the basis of the principle of minimum dissipation, can also be explained in the present framework: this relation emerges as a result of the selection of the fastest-growing mode of the linear instability. The growth velocity of the instability, measured by the quantity Re[*σ*] (>0), is a monotonically increasing function of *q*^2^; the smaller the wavelength, the faster the growth of the instability. However, there exists a minimum wavelength in the present theory; the condition *λ* > *w* should be satisfied, that is, the wavelength of the fastest-growing mode is practically given by 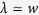. This is because the present theory is valid only when the width of the path is smaller than any other length scales characterizing the shape of the path; the flow is here treated as a (curved) “line”. In this way, the wavelength of the fastest-growing mode 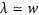 tends to be selected as the wavelength of the meandering path.

##### Phase boundaries

The existence of minimum and maximum values for *w* in the length unit *d*, suggested in the renormalized phase diagram in shown Fig. 2(e), can be explained in the present framework. The maximum value of *w* is given as *λ*_*m*_ from the relations, 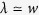 and *λ* < *λ*_*m*_ = (2*π*)^2^*d*/*K*_1_, which were already justified. The minimum of *w* is given by the limitation of the present continuum description: the path interface can be regarded as smooth only when *w* is significantly larger than *d*, that is, the condition *kd* < *w* must be satisfied for the continuum theory with *k* larger than unity. In summary, we expect


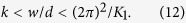


The minimum and maximum of *w*/*d*, *k* and (2*π*)^2^/*K*_1_, should correspond, respectively, to the slopes of the dashed and solid lines (phase boundaries) in Fig. 2(e), which are 3.84 and 11.0, respectively. From this, we obtain 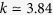 and 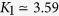, the orders of magnitude of which are consistent with the scaling arguments given above.

This analysis suggests that the solid-like interaction of the form *ρ*_*G*_*gDK*_1_*ζ* employed in Eq. (9) is rather universal and does not depend on the stiffness of the particles. As shown in Fig. 2, the phase boundaries are rather universal for the glass and alumina beads, whereas Young’s modulus of alumina is about five times as large as that of glass. This implies that the value of *K*_1_ is almost the same for the glass and alumina beads despite the large difference in the particle-level stiffness. This may be because the interaction between the air flow and “granular solid” is weak in the sense that it is not related to the deformation associated with the Hertz contact^32^ but rather to the gravity acting on granular materials (note that the term is proportional to *ρ*_*G*_*g*).

## Discussion

Based on our experiment and theory, we have elucidated physical origins of the meandering instability and scale invariance by considering the interaction of the flow with the granular medium, which exhibits dual characters of solid and liquid. (1) Meandering instability occurs for an air flow whose width is fixed by the principle of the floating ping-pong ball. (2) This flow is destabilized due to a linear instability that results from the competition between the centrifugal force (inducing instability) and the restoring force associated with the jammed or solidified granular medium (inducing stability); the flow selects the fastest-growing mode whose wavelength is comparable to the width. (3) The growth of the amplitude of the mode with the selected wavelength is suspended as a result of the competition between the linear instability and the energy dissipation taking place in the granular medium, which is brought about by the fluidized granular medium. The instability tends to increase the amplitude of the path, but when the amplitude becomes larger, the dissipation becomes larger, and the velocity is reduced, thus mitigating the centrifugal force, which is the source of the instability. This subtle balance emerges as the principle of minimum dissipation, which sets the quasi-static amplitude comparable to the width. These mechanisms justify the scale invariance and the phase boundary lines for the meandering regime.

River meandering is a well-known phenomenon and is related in a number of ways to the work described in this paper. Compared with river meandering, the time scale of the meandering instability studied here is extremely small. The present meandering paths frequently disappears, which makes them quasi-static. (This may be due to noise in the flow, possibly created at the exit of the tube; such nose could trigger the instability on its own). Even deep in the meandering regime, the meandering shape is only stable on the sub-second time scale, as suggested in Fig. 4(a) and Supplementary Movies 1–3. In addition, the erosion and accumulation processes, which are crucial to river-meander formation, are considerably different from the processes associated with fluid-solid duality, which is essential to the meandering phenomenon considered in this paper. Nonetheless, the two meandering instabilities share several similarities. Meandering fluids in both cases interact not with an undeformable solid but with a deformable “complex fluid.” In addition, the self-similarity property in Eq. (2) has also been established through field studies of river meandering, and this property seems to be universal and applicable even to meltwater streams on the surfaces of glaciers^26^. Remarkably, the numerical coefficients, *k*_1_ through *k*_3_, found for river meandering are comparable to those found here. We thus expect that the arguments developed here would be useful as a starting point for a physical understanding of river meandering.

This study is also relevant to fluidized beds. Fluidized granular beds have been extensively studied experimentally^33^,^34^,^35^ and numerically^36^,^37^. However, in most cases, air is homogeneously injected at the base of the container, which is quite different from the local injection in the present study. In these homogeneous cases, bubbling has been thoroughly studied. (However, note that even in the less-studied cases in which jets are injected, no attention has been paid to meandering instability^34^,^38^,^39^,^40^). For example, it is known that control of the dynamics of the bubbles created in fluidized reactors could help enhance the efficiency of the reactors^35^,^41^. In the context of fluidized granular beds, the effect of air permeation is very important. The viscous force originating from the interstitial air in the porous medium governs the onset of the bubbling in the bed when the air injection is homogeneous. In contrast, this effect is minor in the present case. This is strongly supported by Figs 2 and 3(c): the discussions associated with the figures relay on Eq. (4), in the derivation of which the flow conservation given in Eq. (8) is assumed. Fluidization processes are essential for industrial applications, such as temperature control, heat transfer, coal combustion and fluidized bed reactors, which are useful for reactions in the conversion of crude oils to gasoline and biomass gasification. The present study is relevant to fluidized beds and might be of some use in such industrial applications.

To compare the present results with those obtained in other contexts, such as geoscience and fluidized beds, it would be useful to discuss these results in terms of more generic dimensionless factors that are introduced from physical pictures at the level of a single particle. (i) In geophysics, the Shields number is quite often used^42^. This number compares (for a grain) the total shear force with the gravitational force, and has been used to discuss the onset of sedimentation. In the present phenomena, a similar number could be defined through the floating condition for a grain (or a “ ping-pong ball”), i.e., the relative importance of the force due to the dynamic pressure compared with the gravitational force. In the present study, this number is a constant of the order of unity in the meandering regime (see the paragraph leading to Eq. (8)). (ii) In the previous study in ref. 27, it is shown that the channel sinuosity, defined as the ratio of the total length along the path to the length projected onto the direction of the average flow, is weakly dependent on the Froude number, which can be regarded as the ratio between the gradient of altitude for a channel in the average-flow direction and the resistance to the flow due to vegetation. The sinuosity values obtained from 20 rivers around the world range from 1.2 to 2.2. In the present case, the sinuosity scales as *R*/*λ*, i.e., the sinuosity is a constant of the order of unity, which is not in conflict with this previous result and is in accordance with the result of the study in ref. 26. (iii) In terms of the Reynolds number for a grain, which is defined as the ratio of the inertial force to the viscous force acting on a grain, in the present study, the fluidized parts of grains are important to the discussion of the dynamic behaviors, and such parts are in the inertial regime, i.e., the Reynolds number is larger than unity, because the interstitial fluid is air in this experiment. However, for the discussion on the solidified part, it is possible that the viscosity of the interstitial air could play a role. (iv) In granular physics, the inertial number has been established to describe a constitutive law for dry granular materials^43^ and the idea is extended to cases with interaction with interstitial fluid^44^,^45^. The same constitutive law could hold for dry and immersed granular materials if the meaning of the inertial number is properly modified. The inertial number for a dry granular material can be regarded as the ratio between an inertial time for rearrangement due to a pressure and a macroscopic time spent by the particle to move from one hole to the next. For the present air flows in a granular medium, the counterpart for the latter time is *d*/*V*. The counterpart for the former rearrangement time may be a time associated with the dynamic pressure or with the gravitational force, and this time scale is given by: 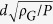 with 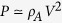 or 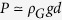, i.e., 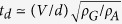 or 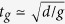, respectively. These times, *t_d_* and *t_g_*, are of the same order of magnitude in the present case because of the floating condition. The single time scale for rearrangements, 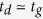, is typically a few ms, which is consistent with the observation made with a high-speed camera (e.g., Supplementary Movie 3). By comparing this rearrangement time scale with the macroscopic time scale, we define the dimensionless number 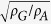, as introduced in the previous studies^44^,^45^. This number would also be useful in comparing the present results with those obtained in the other contexts.

## Conclusion

In this study, we show that an air flow in a thin granular bed can be destabilized to show meandering shape. The phase diagram for the meandering regime is shown in a universal way as a function of a normalized flow rate and a normalized granular bed thickness. In addition, we show that the meandering shapes are self-similar or scale invariant, as observed in river meandering. These experimental results lead to physical insights as summarized in the first paragraph of Discussion.

Fundamentally, our results open a new avenue in the field of granular physics^28^,^46^,^47^ by proposing a minimal description for the fluid-solid duality and a principle of minimal dissipation, leading to a new opportunity for fruitful connections of granular physics to various fields. For example, the physical description in Eq. (9) of the interplay between jamming^48^,^49^ (solid-like response) and fluidization^50^ (dissipation) will be useful in understanding soil-bed fluidization induced by earthquakes^51^, and the present framework, including the principle of minimum dissipation, may help geophysicists to physically understand river meandering. Practically, the present study could be useful for industrial issues such as fluidized-bed reactors.

## Methods

### Experimental

The experiment was recorded using a digital camera (D800E, Nikon, Japan) to obtain the data for analysis. For high-speed visualization of the solidified and fluidized regions, a high-speed camera (UX100, Photoron, Japan) was also used. To both cameras, a macro lens (Micro NIKKOR 60 mm F2.8 ED, Nikon, Japan) was attached. The cell thickness *D* was measured by a laser distance sensor (ZS-HLDS5+ZS-HLDC11+Smart Monitor Zero Pro., Omron, Japan).

### Data Analysis

The measurements of *w*, *A*, *λ*, and *R* are performed as follows. The average value and the error bar (the standard deviation) of *w* and *R* for a single data point in Fig. 3(a–c) are obtained from 30 to 60 measurements. We used 10 to 20 snapshots acquired under the same conditions (the same *ρ*_*G*_, *d*, *D*, and *W*) and selected three points (corresponding to *y* = *nλ* with *n* an integer) from each snapshot to perform the measurements. In each measurement, *R* is determined by fitting a parabolic form to the outside edge of a path. As for *λ* and A* (see Fig. 4(b)), we measured 10 to 20 times for a fixed condition by using 10 to 20 snapshots and making one measurement for each snapshot. In each measurement, *λ* and A* are determined by selecting a segment containing *n* waves (with *n* an integer equal to or larger than 4). To estimate *λ* the length of the segment in the *x* direction is measured, and then divided by the number of waves *n*. To obtain A*, 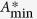 and 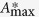 are determined, and then we take the average of these two quantities (We measured A* rather than *A* because the former quantity can be measured in a less ambiguous manner, and then used the relation A* = 2(*A* + *w*/2) to obtain *A*).

### Theory

Details of the linear stability analysis are outlined as follows. Equation (9), combined with *v* = *ζ*_*t*_ + *Vζ*_*x*_, leads to the equation, *ζ*_*tt*_ + 2*Vζ*_*xt*_ + *V*^2^*ζ*_*xx*_ + *κ*_1_*ζ* + *κ*_2_*ζ*_*t*_ = 0, linearized in *ζ*, where *κ*_1_ = *ρ*_*G*_* gK*_1_/(*ρ*_*A*_*w*) and *κ*_2_ = *ρ*_*G*_* gK*_2_*τ*/(*ρ*_*A*_*w*). The solution of the form *e*^*σt*+*iqx*^ with *q* = 2*π*/*λ* satisfies 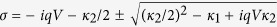. The condition for meandering instability Re *σ* > 0 results in the conditions given in Eq. (10).

## Additional Information

**How to cite this article**: Yoshimura, Y. *et al*. Meandering instability of air flow in a granular bed: self-similarity and fluid-solid duality. *Sci. Rep.* **6**, 38457; doi: 10.1038/srep38457 (2016).

**Publisher's note:** Springer Nature remains neutral with regard to jurisdictional claims in published maps and institutional affiliations.

## Supplementary Material

Supplementary Information

Supplementary Movie 1

Supplementary Movie 2

Supplementary Movie 3
