# Supplementary material for: Meandering instability of air flow in a granular bed: self-similarity and fluid-solid duality
Source: Sci Rep. 2016 Dec 12;6:38457. doi: 10.1038/srep38457 (PMC5150243; doi:10.1038/srep38457)
Supplement: Supplementary Information [file srep38457-s1.pdf]

Description for Supplementary Movies for the article, entitled, "Meandering instability of air flow in a granular bed: self-similarity and fluid-solid duality," by Yuki Yoshimura, Yui Yagisawa, and Ko Okumura

Supplementary Movie 1

The air flow in a vertical granular bed, showing quasi-static meandering shapes for Q = 75 ml/min, W = 80 mm, and D = 1.0 mm. The play speed is one half of the real one.

Supplementary Movie 2

The air flow, showing unstable and turbulent shapes for Q = 50 ml/min, W = 80 mm, and D = 1.0 mm. The play speed is also one half.

Supplementary Movie 3

The air flow, exhibiting the solid-fluid duality along the meandering path. This is a high-speed movie (4000 fps) of a meandering path for Q = 100 ml/min, W = 80 mm, and D = 1.0 mm (The play speed is 100 times slower than the real one).
